# CHA2DS2-VASc Is Associated With In-Hospital Mortality in Patients With Infective Endocarditis: A Cross-Sectional Cohort Study

**DOI:** 10.7759/cureus.11620

**Published:** 2020-11-22

**Authors:** Temidayo Abe, Gabrielle De Allie, Harry O Eyituoyo, Tolulope Abe, Temitope Tobun, Jennifer C Asotibe, Dolphurs Hayes, Paul Mather

**Affiliations:** 1 Internal Medicine, Morehouse School of Medicine, Atlanta, USA; 2 Internal Medicine/Community Medicine, Mercer University School of Medicine, Macon, USA; 3 Internal Medicine, All Saints University School of Medicine, Roseau, DMA; 4 Medicine, John H. Stroger, Jr. Hospital of Cook County, Chicago, USA; 5 Department of Medicine, Morehouse School of Medicine, Atlanta, USA; 6 Department of Cardiovascular Disease, Perelman School of Medicine, Philadelphia, USA

**Keywords:** chad vasc score, infective endocarditis, national inpatient sample

## Abstract

Background and objective

The CHA_2_DS_2_-VASc score is a stroke risk stratification tool that is used in patients with atrial fibrillation (AF). Most of its clinical variables have been associated with poor outcomes in patients with infective endocarditis (IE). In this study, we aimed to determine its utility in predicting outcomes in IE patients.

Methods

We included 35,570 patients with IE from the National Inpatient Sample (NIS), 2009-2012. The CHA_2_DS_2_-VASc score was calculated for each patient. Hierarchical logistic regression was used to estimate the adjusted odds ratio for in-hospital mortality for CHA_2_DS_2_-VASc scores from 1 to 9, using a score of 0 as the reference score. All clinical characteristics were defined using the International Classification of Diseases, Ninth Revision, Clinical Modification (ICD-9-CM) codes.

Results

The mean age of the sample was 57.81 ±14 years. Higher CHA_2_DS_2_-VASc scores were associated with increased mortality, and the scores among the sample ranged from 0 for 8.1% to 8 for 21.7%. In the hierarchical logistic regression, after adjusting for age, sex, and relevant comorbidities, as the score increased, so did the odds for overall mortality.

Conclusion

In patients with IE, the CHA_2_DS_2_-VASc score may serve as a risk assessment tool with which to predict outcomes. Further studies are needed to replicate these findings.

## Introduction

Infective endocarditis (IE) is a life-threatening infection of the valvular or paravalvular heart structures; it has an incidence rate of approximately 100 cases per one million patient-years [1,2]. Although it is a relatively uncommon disease, the in-hospital mortality rate associated with it can be as high as 20% [3]. Also, it carries significant long-term mortality and morbidity risks, with a high rate of stroke (24%), heart failure (49%), and sudden cardiac death (11%) [1,3]. Despite several ongoing advancements in the medical field, optimal IE management still remains a challenge [2]. One of the significant challenges associated with IE management is appropriately identifying and stratifying patients based on the increased risk of complications [2]. A scoring system that predicts complications may help with early risk stratification and determine who will benefit from further interventions.

The CHA_2_DS_2_-VASc score was created to predict stroke risks in patients with atrial fibrillation (AF) [4,5]. While it was initially limited to patients with AF, further studies have demonstrated its usefulness in predicting outcomes in patients without AF. In one study involving elderly patients, the CHA_2_DS_2_-VASc score was able to predict ischemic strokes and transient ischemic attacks in patients with or without AF [6]. In another study, it was used to predict major cardiovascular events in patients undergoing percutaneous coronary interventions [7]. More recently, Guido et al. have demonstrated the utility of the CHA_2_DS_2_-VASc score in predicting cardiovascular events and long-term outcomes in patients with Takotsubo cardiomyopathy [8].

The CHA_2_DS_2_-VASc scoring system incorporates a history of congestive heart failure, hypertension, diabetes, prior stroke, vascular disease, patient age, and sex [9]. Some of the clinical entities in this risk assessment tool have been associated with poorer outcomes in patients with IE. Firstly, diabetes mellitus, which doubles the in-hospital mortality rate, is believed to play an important role due to hyperglycemia’s adverse effects on the immune system [3]. Furthermore, patient age, female gender, and congestive heart failure have also been associated with poor outcomes [3,10-12]. In one study, Thuny et al. demonstrated that premorbid heart failure, patient age, and female gender were strongly associated with the risk of embolism and death in IE based on an echocardiographic evaluation. According to their findings, the relative risks of one-year mortality for IE with comorbid heart failure, female gender, and age were 1.9, 1.8, and 1.03, respectively (p=0.005, 0.009, and 0.0003, respectively) [12]. Based on these findings, we aim to investigate the association between the CHA_2_DS_2_-VASc score and in-hospital mortality among IE patients.

## Materials and methods

Study design

This was a cross-sectional study. The data related to the study cohort were collected from the National Inpatient Sample (NIS) database, 2009-2012, a subset of the Healthcare Cost and Utilization Project (HCUP) sponsored by the Agency for Healthcare Research and Quality (AHRQ). NIS is the largest all-payer inpatient database in the US. It contains a 20% stratified sample of all discharges from US nonfederal, short-term general hospitals, subspecialty hospitals, and public hospitals. It is stratified based on the number of beds, ownership, hospital teaching status, US region, and state. Stratified random sampling ensures that the database is representative of the US population and accounts for 90% of all hospitalizations in the US after applying appropriate weights. The NIS includes information on demographic characteristics, hospital characteristics, up to 25 diagnostic and procedure codes based on the International Classification of Diseases, Ninth Revision, Clinical Modification (ICD-9-CM) codes, and outcomes based on patient discharge records. Each record represents a single hospitalization, and thus, multiple records may exist for an individual with recurrent hospitalizations. The details regarding the NIS are freely available online.

Study population, variables, and outcomes

The study group consisted of patients hospitalized between 2009 and 2012 and included in the NIS database, aged 18 years and above, with a primary diagnosis of IE (n=35,570). The CHA_2_DS_2_-VASc score was calculated for each patient using the following variables: a history of congestive heart failure, hypertension, diabetes, prior stroke, vascular disease, age (<65 years: score 0; 65-74 years: score 1; and >75 years: score 2), and sex (female: score 1; male: score 0). A history of congestive heart failure, hypertension, diabetes, prior stroke, and vascular disease were all identified in any of the 25 available diagnosis codes in the NIS using their respective ICD-9 codes. In addition, NIS variables were used to identify patients’ demographic characteristics, such as age and gender. Other variables that could impact study outcomes, such as a history of endocarditis, hemodialysis, and valve replacement, were also included (Table 1). The outcome of interest was all-cause in-hospital mortality, which was defined as death due to any cause during a hospital stay.

Statistical analysis

IBM SPSS Statistics version 25 (IBM, Armonk, NY) was used for the statistical analysis. A hierarchical two-level logistic regression model, with hospital ID as a random effect, was used to evaluate the outcome variables. We quantified the predictive validity of the classification schemes by using a two-level model to test the hypothesis that these classification schemes performed significantly better than chance. A study cohort with a CHA_2_DS_2_-VASc score of 0 was used as the reference score. A p-value of <0.05 was considered to be statistically significant.

## Results

**Figure 1:**
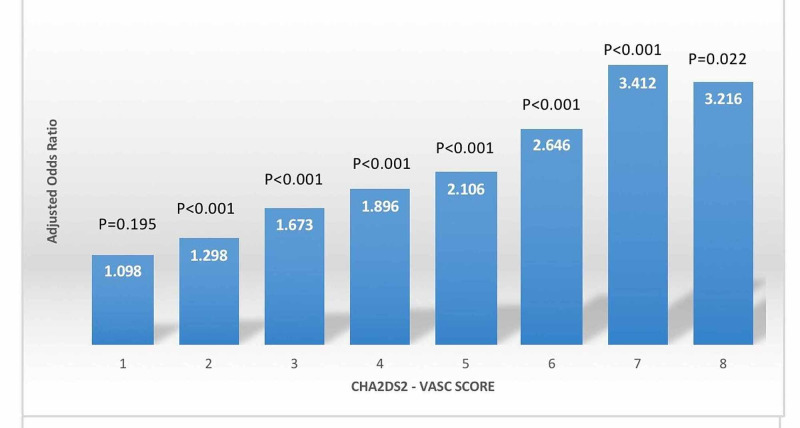
Graphic representation of the adjusted odds ratio for in-hospital mortality for CHA2DS2-VASc scores 1-9, using score 0 as the referent score

We identified 35,570 patients with a primary diagnosis of IE from the NIS. The mean age of the sample was 57.81 ±14 years. The most prevalent score among the sample was 2 (22.8%, 8,105/35,570), while a score of 8 (0.1%, 23/35,570) was the least prevalent. The baseline characteristics and comorbidities contributing to the CHA_2_DS_2_-VASc score are listed in Table 1. For the 35,570 patients with IE, the in-hospital mortality rate was 11.4% (n=4,038/35,570). A higher CHA_2_DS_2_-VASc score was associated with poorer outcomes in patients with IE. In-hospital mortality increased from 8.1% for a score of 0 to 21.7% for a score of 8 (Table 1). The hierarchical logistic regression showed a similar trend. A higher score was significantly associated with increased odds of in-hospital mortality. Using a score of 0 as the referent score, the odds ratios for overall mortality increased from 1.30 (95% CI: 1.13-1.49, p<0.001) for a score of 2 to 3.22 (95% CI: 1.18-1.49, p=0.022) for a score of 8 (Figure 1, Table 2).

**Table 1:** Baseline patients characteristics CHF: congestive heart failure; COPD: chronic obstructive pulmonary disease; CKD: chronic kidney disease; TIA: transient ischemic attack; VHD: valvular heart disease; HIV: human immunodeficiency virus

Demographics	CHA_2_DS_2_-VASc score								
	0	1	2		4	5	6	7	8
Overall, n	3,753	7,646	8,105	7,324	5,383	2,391	779	166	23
Age									
<65 years, %	18.8	34.5	26.5	13.4	5.3	1.2	0.2	0.0	0
65-74 years, %	0	11.4	28.9	31.8	18.5	6.7	2.2	0.4	0
>75 years, %	0	0	9.8	28.2	34.5	19	6.7	1.6	0.2
Gender									
Female, %	0	18.7	21	21.9	21.2	11.6	4.4	1.1	0.2
Male, %	100	64.5	62.5	56.5	43	29.6	17.7	0.1	0
Comorbidities									
Hypertension, %	0	14.2	23.7	24.8	22	10.9	3.6	0.8	0.1
Diabetes, %	0.0	13.8	26.7	26.1	19	9.4	3.8	0.9	0.3
CHF, %	0	6.7	19.7	25.6	26.4	15.8	4.4	1.2	0.2
Stroke or TIA, %	0	0	13.2	25.4	26.2	18.4	12.6	3.8	0.5
VHD, %	0	2.8	14.9	23.9	27.6	19.8	8.8	1.6	0.5
Obesity, %	4.5	17.9	27.2	22.5	17.6	7.3	2.5	0.5	0.1
COPD, %	4.4	13.9	22	25.3	21.0	9.9	2.7	0.7	0.1
CKD, %	1.9	16.5	26.2	23.7	19.1	9.1	2.7	0.6	0.1
History of endocarditis, %	5.0	20	2	2	21	3	2	0	0
History of valve replacement, %	9.7	20.1	23.4	20.4	16.1	7.4	2.5	0.3	0.1
HIV, %	25.7	33.4	24.8	11.6	3.2	1.1	0.2	0	0
Hemodialysis, %	1.4	18.8	28.8	24	17	7.8	1.7	0.3	0.1
Outcome									
Overall mortality, %	8.1	8.8	10.2	12.7	14.1	15.5	18.7	23	21.7

**Table 2:** Adjusted odds ratio for each outcome based on CHA2DS2-VASc score This model was adjusted for age, sex, and comorbidities being significantly different among the patient population (p<0.05)

In-hospital mortality				
		95% confidence interval		
	Odds ratio	Lower limit	Upper limit	P-value
CHA_2_DS_2_-VASC score				
0	Referent	Referent	Referent	
1	1.098	0.953	1.266	0.195
2	1.298	1.13	1.49	<0.001
3	1.673	1.459	1.918	<0.001
4	1.896	1.647	2.184	<0.001
5	2.106	1.79	2.478	<0.001
6	2.646	2.132	3.284	<0.001
7	3.412	2.33	4.996	<0.001
8	3.216	1.185	8.733	0.022

## Discussion

In this study, we investigated the utility of the CHA_2_DS_2_-VASc score as a risk assessment tool in predicting in-hospital IE outcomes. Our findings demonstrated that CHA_2_DS_2_-VASc is associated with in-hospital mortality among IE patients. To our knowledge, this is the first study to evaluate the role of the CHA_2_DS_2_-VASc score in IE.

IE is associated with a wide variety of complications involving various organ systems. Cardiac complications range from congestive heart failure to myocardial abscess and valvular perforation [13]. Neurological complications include brain abscess, meningitis, and cerebral hemorrhage [12]. Systemic embolism can occur, especially in patients with left-sided IE, which correlates to the vegetation's size, which can result in glomerulonephritis in the kidneys and pulmonary embolism in the lungs [12,14-16].

Current IE management strategies are based on presumed patient risk. Low-risk patients can be safely managed with antibiotics, while early aggressive interventions, such as cardiac surgery, are recommended for those with a high risk of mortality [17]. These include those with heart failure or severe valve dysfunction, large vegetation (>10 mm in diameter), mechanical complications such as valve perforation, dehiscence, abscess cavities, or a new heart block, persistent bacteremia with highly resistant organisms, or fungal endocarditis [17,18]. These recommendations are based on observational studies demonstrating decreased mortality with surgical interventions in these patient populations [19-21]. The challenge now lies in identifying other high-risk patients who will benefit from surgical interventions, especially at an earlier stage during their hospitalizations [2,22].

A scoring system that leads to adequate patient selection, bedside decision-making, and patient education and provides prognostic information may help decrease IE-associated mortality. There are currently no validated scoring models to predict in-hospital complications or outcomes of IE. Current models have focused on long-term outcomes and complications after cardiac surgery [23-25]. As demonstrated in this study, the CHA_2_DS_2_-VASc score may serve as a risk assessment tool in patients with IE. In our study, patients with a score of 2 or more had mortality rates greater than 10%. In-hospital mortality rates increased as the score increased, from 12% for a score of 2 to 21.7% for a score of 8. The odds of in-hospital mortality doubled at a score of 2. It increased by 20% for a score of 2 as compared to a score of 1, and it further increased by 38% for a score of 3 as compared to a score of 2. This suggests that this model may be used to risk-stratify patients with IE. Patients with high scores (e.g., >1) may benefit from early surgery and closer monitoring.

Prognosis in IE patients is driven by patient characteristics, the presence or absence of cardiac complications, infecting organisms, and echocardiographic features [17]. Patients with more underlying comorbidities (i.e., older age, diabetes mellitus, and cardiac/pulmonary/renal diseases) tend to have poorer outcomes [3,17]. In this study, we found increased odds for in-hospital mortality with an increase in the CHA_2_DS_2_-VASc score. One potential explanation is that a higher level of comorbidity is associated with a higher score. Another plausible explanation is that the scoring system incorporates certain specific disease conditions (i.e., age, female sex, diabetes mellitus, and congestive heart failure) that have been associated with poorer outcomes in patients with IE, as we hypothesized. One limitation of using this scoring system would be the absence of important clinical variables, such as echocardiographic features and organisms involved, which have been linked to poor prognosis in patients with IE.

Our study has some limitations related to the NIS database. Firstly, the NIS is an administrative database fraught with variations in institutional coding practices; hence, it is susceptible to coding errors. Secondly, we had no data on clinical variables such as echocardiographic features, vegetation size, vital signs, and the organisms involved, all of which have a significant impact on the rates of complications [3,26]. Also, data on antimicrobials and the duration of treatment could not be obtained, and this may have had an impact on study outcomes [26,27].

## Conclusions

In conclusion, the CHA_2_DS_2_-VASc score may help with risk-stratifying patients with IE. Further studies are needed to replicate this finding and to investigate how the tool can be implemented in in-patient management and bedside decision-making.
